# Corticosteroid Dosing Level, Incidence and Profile of Bacterial Blood Stream Infections in Hospitalized COVID-19 Patients

**DOI:** 10.3390/v16010086

**Published:** 2024-01-05

**Authors:** Ivan Papic, Petra Bistrovic, Tomislav Cikara, Nikolina Busic, Tatjana Keres, Maja Ortner Hadziabdic, Marko Lucijanic

**Affiliations:** 1 Pharmacy Department, University Hospital Dubrava, 10000 Zagreb, Croatia; 2 Cardiology Department, University Hospital Dubrava, 10000 Zagreb, Croatia; 3 Department of Internal Medicine, University Hospital Dubrava, 10000 Zagreb, Croatia; 4 Centre for Applied Pharmacy, Faculty of Pharmacy and Biochemistry, University of Zagreb, 10000 Zagreb, Croatia; 5 Hematology Department, University Hospital Dubrava, 10000 Zagreb, Croatia; 6 School of Medicine, University of Zagreb, 10000 Zagreb, Croatia

**Keywords:** corticosteroid dose, COVID-19, bacteremia, blood stream infection, dexamethasone

## Abstract

COVID-19 patients with severe or critical symptoms are often treated with corticosteroids, per contemporary guidelines. Due to their immunosuppressive and immunomodulatory properties, corticosteroids are associated with the development of superinfections. We aimed to retrospectively assess patterns of corticosteroid use and the profiles of bacterial blood stream infections associated with exposure to different dosing levels, in a cohort of 1558 real-life adult COVID-19 patients. A total of 1391 (89.3%) patients were treated with corticosteroids, with 710 (45.6%) patients receiving low, 539 (34.6%) high and 142 (9.1%) very high corticosteroid doses. Bacteremia developed in a total of 178 (11.4%) patients. The risk of bacteremia was of similar magnitude between the no and low-dose corticosteroid treatments (*p* = 0.352), whereas it progressively increased with high (OR 6.18, 95% CI (2.66–14.38), *p* < 0.001) and very high corticosteroid doses (OR 8.12, 95% CI (3.29–20.05), *p* < 0.001), compared to no corticosteroid treatment. These associations persisted after multivariate adjustments and were present independently of sex, comorbidity burden, and mechanical ventilation. The profiles of individual bacterial pathogens differed depending on the used corticosteroid doses. High and very high corticosteroid doses are frequently used for real-life COVID-19 patients with severe and critical clinical presentations and are associated with a higher risk of bacteremia independently of sex, comorbidity burden, and mechanical ventilation use.

## 1. Introduction

Coronavirus disease 2019 (COVID-19) may be clinically presented in a range from asymptomatic infection to severe pneumonia and critical disease, accompanied by acute respiratory distress syndrome (ARDS) [1]. Histopathological lung samples from patients who died from COVID-19 found microvascular thrombosis, diffuse alveolar damage, and inflammatory cell infiltration [2]. The acknowledged pathophysiologic mechanism responsible is the neutrophil-generated hyperinflammatory response with an increased production of inflammatory cytokines: interleukin-1, interleukin-6 (IL-6), and tumor necrosis factor, resulting in a cytokine storm [3]. To alleviate the hyperactive immune system activation and improve clinical outcomes in severe or critical COVID-19 patients, the use of corticosteroids (CS), IL-6 receptor blockers, and Janus kinase inhibitors are recommended by the World Health Organization (WHO) [4]. Infection is a well-known corticosteroid adverse drug reaction. The proposed mechanism is related to its pharmacologic action of inhibiting macrophage and dendritic cell differentiation, as well as inhibiting T-cell activation. Simultaneously, they inhibit the production of interleukins and tumor necrosis factor, along with inflammatory prostaglandins [5]. Modifying the balance of immune system corticosteroids might promote the development of blood stream infections (BSIs).

In hospitalized COVID-19 patients, a high incidence of bacteremia has been observed by various reports [6,7,8,9,10]. BSIs in this population are associated with lower oxygen saturation, more frequent respiratory failure requiring mechanical ventilation (MV), the development of septic shock, intensive care unit (ICU) admissions, longer hospitalizations, and higher mortality [11,12,13]. Glucocorticoids are the first and the most often used drugs, with immunosuppressive and immunomodulatory properties, recommended for the treatment of severe and critical forms of the disease [4,14]. Although their use in the aforementioned group of patients is well-established, the optimal glucocorticoid dose and duration of the treatment, regarding efficacy and safety, remain inconclusive, especially when questioning the use of higher doses [15,16,17,18].

The use of high-dose glucocorticoids were associated with a high risk of BSIs in various clinical scenarios among immunocompromised patients [19,20,21,22]. Furthermore, higher doses and an earlier commencement of corticosteroid treatment significantly increased the odds of bacteremia in influenza-related ARDS patients [23]. The association between corticosteroid use in COVID-19 patients and bacteremia varied considerably [24,25]. Due to these stated uncertainties, in our research, we aimed to assess patterns of corticosteroid dosing level with the incidence and profiles of bacterial blood stream infections in a cohort of hospitalized COVID-19 patients.

## 2. Materials and Methods

### 2.1. Patients and Study Design

We retrospectively analyzed a cohort of 1558 COVID-19 patients with either severe or critical symptoms, who were hospitalized in a tertiary level referral center from March 2020 to June 2021. All patients were >18 years old and were Caucasians. All patients tested positive for SARS-CoV-2, either on polymerase chain reaction (PCR) or antigen tests, in the presence of compatible clinical symptoms. The inclusion criteria were hospital admission to our center due to COVID-19, infection proven by PCR or antigen test, and available written medical records for the evaluation of daily corticosteroid dosing. No specific exclusion criteria were applied, besides not meeting the inclusion criteria.

COVID-19 severity was determined using the World Health Organization (WHO) recommendations. Patients were treated according to the contemporary WHO guidelines, with the majority receiving corticosteroids (89.3%) and low molecular weight heparin (LMWH, 95.9%), while 50% received remdesivir. None of the patients received tocilizumab, baricitinib, or other anti-cytokine therapy. Data on demographic and clinical characteristics and the occurrence of bacteremia were obtained by the analysis of written and electronic medical records for each patient individually. Comorbidities were evaluated as a cumulative comorbidity burden, assessed using the Charlson comorbidity index (CCI). BSI, i.e., bacteremia, was considered in the case of positive blood cultures taken, based on the clinical reasoning of the treating physicians. Single blood cultures with isolates of contaminants such as Coagulase-negative *Staphylococcus* (CoNS) and *Corynebacterium species* were excluded. We defined a real BSI caused by skin commensals as at least two consecutive blood culture sets of the same species and a clinical course consistent with BSI. Corticosteroid dosing level was graded based on a maximal used daily dose of a particular drug, translated into the prednisone equivalent dose, rather than the cumulative or average dose used. This reasoning was based on the fact that higher corticosteroid doses were mainly given based on the clinical reasoning of treating physicians, and higher doses were mostly guided by the deteriorating state of the patient. Since clinical decision-making was based on a day-to-day basis, we consider maximal daily doses to most accurately represent the intended dose strength. Doses in the range of 1–2 mg/kg body weight are considered therapeutic doses for a range of medical conditions requiring immunosuppression, and similar cut-offs were used in previous papers investigating corticosteroid doses [26,27]. Thus, patients in the current study were classified based on maximal daily corticosteroid dose given during their hospital stay, as no corticosteroid use, low doses (below 1 mg/kg body weight), high doses (1–2 mg/kg body weight), and very high doses (above 2 mg/kg body weight).

### 2.2. Ethical Approval

The study was approved by the University Hospital Dubrava Review Board (Nm. 2021/1410-01). The study was conducted following the Declaration of Helsinki.

### 2.3. Statistical Analysis

Categorical variables were presented as frequencies and percentages and were compared between subgroups using the chi-squared test and the chi-squared test for trend. Numerical variables were evaluated for the normality of distribution using the Kolmogorov–Smirnov test. Due to their non-normal distribution, they were presented as median and interquartile range (IQR). Odds ratios (OR) and adjusted ORs with respective 95% confidence intervals (CI) for bacteremia occurrence between categories of corticosteroid dosing levels were obtained using logistic regression. *p* values < 0.05 were considered statistically significant. All analyses were done using the MedCalc statistical program, version 22.007 (MedCalc Software Ltd., Ostend, Belgium).

## 3. Results

### 3.1. Overview of Patient Characteristics, Corticosteroid Dosing Level, and Bacteremia

A total of 1558 patients were analyzed. There were 966 (62%) male and 592 (38%) female patients. The median age was 66 years (IQR: 57–74). The median CCI was three points (IQR: 2–4). An overview of baseline patient comorbidities is provided in Table 1. A total of 1262 (81%) patients presented with severe and 270 (17.3%) with critical COVID-19 symptoms at hospital admission, and ultimately all patients developed a severe or critical form of the disease during hospitalization.

A total of 1391 (89.3%) patients were treated with corticosteroids, with 710 (45.6%) patients receiving low, 539 (34.6%) high, and 142 (9.1%) very high corticosteroid doses. The most frequently used drugs were dexamethasone by 1041 (66.8%), followed by methylprednisolone by 674 (43.3%), and prednisone by 66 (4.2%) patients. Corticosteroids were started on the median 1st day of hospitalization (IQR: 1st–1st). The median duration of corticosteroid treatment was 10 days (IQR: 6–13).

BSI were present in a total of 178 (11.4%) patients. Gram-positive, Gram-negative, and simultaneous Gram-positive and Gram-negative BSI were present in 90 (5.8%) and 128 (8.2%) patients, respectively, whereas polymicrobial BSI were present in 79 (5.1%) patients. The most frequently isolated bacterial pathogens were *Acinetobacter baumannii* in 96 (6.2%), CoNS (after the exclusion of contaminates) in 37 (2.4%), *Staphylococcus aureus* in 31 (2%), *Enterococcus faecalis* in 24 (1.5%), *Klebsiella pneumoniae* in 23 (1.5%) and *Enterococcus faecium* in 15 (1%), whereas other individual pathogens were isolated in <1% of all investigated patients.

### 3.2. The Association of Corticosteroid Dosing Level with the Occurrence of Bacteremia

**Figure 1:**
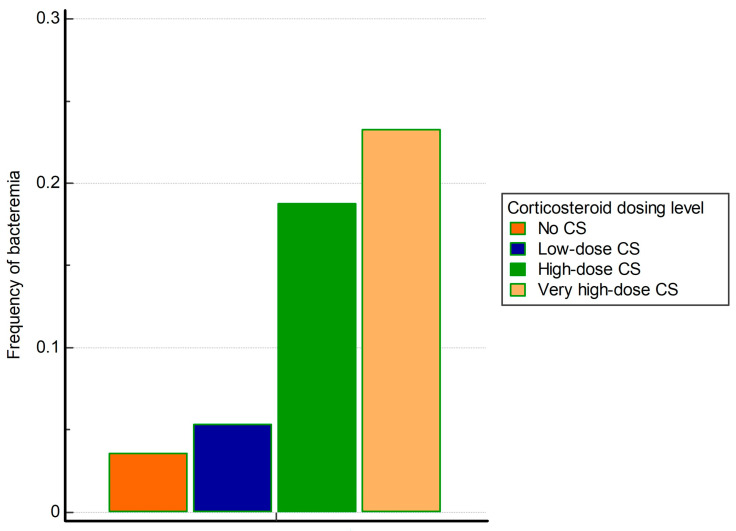
Frequency of bacteremia regarding corticosteroid dosing level.

Corticosteroid dosing level was significantly associated with the occurrence of BSI (*p* < 0.001). As shown in Figure 1, bacteremia was present in 6 (3.6%) patients without corticosteroid treatment, 38 (5.4%) treated with low, 101 (18.7%) high, and 33 (23.2%) very high corticosteroid dose. The risk of bacteremia was of similar magnitude between no and low-dose corticosteroid treatment (*p* = 0.352), whereas it progressively increased with high (OR 6.18, 95% CI (2.66–14.38), *p* < 0.001) and very high corticosteroid doses (OR 8.12, 95% CI (3.29–20.05), *p* < 0.001), compared to no corticosteroid treatment.

This association persisted after multivariate adjustments, and high (aOR 1.85, 95% CI (1.21–2.82), *p* = 0.004) and very high corticosteroid doses (aOR 1.96, 95% CI (1.12–3.44), *p* = 0.017), in comparison to no corticosteroid treatment, remained significantly associated with a higher risk of BSI, independently of male sex (aOR 1.73, 95% CI (1.16–2.58), *p* = 0.008), lower comorbidity burden (aOR 0.88, 95% CI (0.79–0.99), *p* = 0.046), and the need for mechanical ventilation (aOR 18.9, 95% CI (11.9–30.03), *p* < 0.001), as shown in Table 2. The model was created through a backward selection process, additionally considering active malignancy, active chemotherapy, and the presence of infections other than COVID-19 at admission.

We further separately analyzed remdesivir-non-exposed and remdesivir-exposed patients. The corticosteroid dosing level was significantly associated with a higher occurrence of BSI in unadjusted analyses, in both remdesivir-non-exposed and -exposed patients (*p* < 0.001 for both analyses). A multivariate analysis among remdesivir-non-exposed patients recognized similar independent predictors of BSI as those observed in the entire cohort, including high (aOR 4.16, 95% CI (1.18–14.64), *p* = 0.026) and very high corticosteroid doses (aOR 7.21, 95% CI (1.81–28.63), *p* = 0.005) and the need for mechanical ventilation (aOR 19.21, 95% CI (9.63–38.32), *p* < 0.001). Among remdesivir-exposed patients, corticosteroid dosing level did not independently contribute to the occurrence of BSIs, whereas male sex (aOR 1.88, 95% CI (1.08–3.28), *p* = 0.026), a lower comorbidity burden (aOR 0.81, 95% CI (0.68–0.97), *p* = 0.024), and the need for mechanical ventilation (aOR 19.44, 95% CI (10.42–36.26), *p* < 0.001) were recognized as independent predictors of BSI.

### 3.3. Profile of Bacterial Blood Stream Infections Regarding the Corticosteroid Dosing Level

The profiles of bacterial blood stream infections, regarding corticosteroid dosing level, are shown in Table 3.

A higher corticosteroid dosing level was significantly associated with higher occurrences of Gram-positive, Gram-negative, and polymicrobial bacterial blood stream infections (*p* < 0.001 for all analyses). A higher corticosteroid dose was significantly associated with a higher incidence of blood stream infections with a large number of pathogens (*Acinetobacter baumannii*, *Staphylococcus aureus*, *Enterococcus faecalis*, *Coagulase negative Staphylococcus*, *Corynebacterium species*, *Stenotrophomonas maltophilia*, and *Beta hemolytic Streptococcus*), but a lower incidence of bacteremia caused by *Proteus mirabilis* (*p* < 0.05 for all analyses).

Similar incidences of bacteremia caused by *Enterococcus faecium*, *Klebsiella pneumoniae*, *Pseudomonas aeruginosa*, *Escherichia coli*, *Klebsiella aerogenes*, *Enterobacter cloacae*, *Staphylococcus haemolyticus*, *Serratia marcescens*, *Providencia stuartii*, *Streptococcus pneumoniae*, *Bacteroides species*, *Haemophilus parainfluenzae*, and *Providencia rettgeri* were observed, regardless of the used corticosteroid dose (*p* > 0.05 for all analyses).

## 4. Discussion

To our knowledge, our study is the first one reporting on the incidence of bacteremia and its association with corticosteroid doses used to treat COVID-19 patients. As our data demonstrate, there is a consistent association between the use of high and very high CS doses and BSI, whereas there was a similar risk of BSI between patients without and those with low-dose CS. An increase in bacteremia frequency was present among almost all frequently isolated pathogens. Glucocorticoids are used to alleviate inflammation and suppress the immune system. Their use comes with a greater risk of secondary infections. The risk is particularly increased in patients receiving high corticosteroid doses [21,22,28], and our study is the first to demonstrate this phenomenon in COVID-19 patients.

An earlier report from Giacobbe et al. turned researchers’ attention to the association between the use of steroids and other immunomodulatory therapy, with a higher risk of BSI among ICU patients. The following studies suggested more opposite than confirmative findings, leaving possible associations inconclusive [10,24,29,30,31,32]. Studies described the association between the use of glucocorticoids and the occurrence of BSI, but no study explored the association between dose strength and BSI, on which we report here. The RECOVERY trial established the use of corticosteroids, namely 6 mg of dexamethasone daily, as a standard of care for severe COVID-19 patients receiving respiratory support, due to the subsequent improvement in mortality [14]. Later research found no substantial mortality benefit when using doses of 20 mg or 12 mg dexamethasone [33,34,35]. In the COVID STEROID 2 trial, the overall recorded occurrence of bacteremia was low in both 12 mg and 6 mg dexamethasone treatments, at 0.8% and 0.2%, respectively. In our study, two mentioned doses would have been considered low-dose [34]. Although there are no data regarding previous CS doses available, we speculate that the majority of patients in previous studies were also treated with low steroid doses, as recommended by contemporary guidelines [4]. In our research, almost half of the patients (45.6%) of the whole cohort were treated with low-dose glucocorticoid, and their risk of bacteremia was comparable to those patients without treatment (*p* = 0.352), which supports the majority of the findings of earlier research.

Our real-life data, based on mostly elderly and comorbid patients with severe and critical COVID-19, show that nearly half of the patients (48.9%) treated with CS received high and very high doses (38.7% and 10.2%, respectively). The utilization of high corticosteroid doses increased the risk of bacteremia six times, and very high doses increased it by eight times, compared to no corticosteroid treatment. Moreover, a multivariate logistic regression analysis showed that the association of high and very high glucocorticoid doses and BSI persisted independently of the male sex, a lower comorbidity burden, and the need for mechanical ventilation, although the magnitude of the effect was alleviated when concomitantly considering these confounders. It should be noted that the association of BSI with a lower comorbidity burden should be interpreted only in the context of other factors included in the multivariate analysis (corticosteroid dosing level, sex, and mechanical ventilation) that share the prognostic contribution of the CCI to the risk of BSI. Otherwise, a higher comorbidity burden was shown to contribute to the higher incidence of bacteremia [10,25,31,32,36].

Previous studies that focused on BSI did not report the doses of CS used, and studies evaluating higher CS doses lacked data regarding bacteremia occurrence. Of 12 studies included in the meta-analysis, evaluating high and very high-dose versus low-dose glucocorticoids in COVID-19 patients, none reported the incidence of BSIs [17]. Two mentioned comparable incidences of bacterial infection, while one reported secondary infections being more common in the high-dose group (24 mg of dexamethasone) [37,38,39]. The randomized control trial CODEX is the only study reporting bacteremia data. The use of 20 mg dexamethasone for 5 days, followed by 10 mg dexamethasone, did not significantly increase the risk of bacteremia compared to the standard of care (7.9 vs. 9.5%); neither did the development of secondary infection [33]. In our trial, the doses used in CODEX would have been considered high doses. Although the high doses used in CODEX trial were not associated with a higher infection rate, safety still remains the main concern, especially in real-life patients treated with very high CS doses.

The overall occurrence of bacterial bacteremia in our cohort was 11.4%. Previous studies reported various incidences of bacterial BSI, ranging from as low as 1% up to 5% in general and non-ICU patient cohorts [10,12,29,40,41]. An ICU stay multiplied the risk of bacteremia from 15 to 49% of the patients [18,24,25,32,42,43], consistent with our findings of mechanical ventilation increasing the risk.

The most frequent isolated bacterial pathogens in our cohort were *Acinetobacter baumannii* (6.2%) followed by Gram-positive cocci, namely *Enterococcus species* (3%), CoNS (2.4%), and *Staphylococcus aureus* (2%), ending with *Klebsiella pneumoniae* (1.5%). Of those positive blood culture isolates, a higher occurrence of *Acinetobacter baumannii*, *CoNS*, *Staphylococcus aureus* and *Enterococcus species* was significantly associated with the use of higher corticosteroid doses (*p* < 0.05 for all analyses), while the occurrence of *Klebsiella pneumoniae* isolates was comparable. *Acinetobacter baumannii* was reported in earlier studies as the most frequent Gram-negative BSI in COVID-19 patients [32,44]. Our findings are very similar to those of Frattari et al., who found that not only was *Acinetobacter baumannii* the most frequent cause of bacteremia, while preceded colonization was documented in only 7% of cases, but that bacteremia was implied to be hospital-acquired [36]. Moreover, the use of corticosteroids was found to be associated with hospital-acquired *Acinetobacter baumannii* bacteremia, resulting in poorer outcomes, while early administration was associated with increased bacterial resistance [44,45]. These, along with previous findings of an association between high-dose steroids and *Acinetobacter* bacteremia [46], support our hypothesis.

Increases in *Enterococcus* BSIs were frequently reported in COVID-19 patients, and more frequently than in the non-COVID population [6,7,12,32,43,44]. Genome sequencing performed in one study advocates against nosocomial transmission [43]. Whether the increase in *Enterococcal* bacteremia is due to systemic inflammation, or gut translocation due to the microbiome dysbiosis observed in COVID-19 patients, frequent concomitant use of ceftriaxone, or some still unknown mechanism responsible, remains to be explored [47,48]. Nosocomial infections with resulting *CoNS* bacteremia have been previously described, and they exhibited similarities to the findings in our study [8,30]. Higher rates of *Staphylococcus aureus* and its mortality in COVID-19 patients were also previously reported. Nosocomial transmission may be due to known risk factors, such as intubation and central venous catheters [8,9,49,50]. Interestingly, although the frequency of *Klebsiella pneumoniae* bacteremia was notable, its occurrence did not increase along with the CS dose. This exception is hardly explainable, since data on *Klebsiella pneumoniae* BSI in COVID-19 patients are scarce.

The use of remdesivir has been associated with a higher BSI occurrence in COVID-19 patients [51,52]. We separately evaluated remdesivir-non-exposed and -exposed sub-cohorts and observed the loss of CS predictive properties among remdesivir-treated patients, after controlling the analyses for other clinically meaningful parameters. These results are in line with previous observations, as remdesivir use might increase the occurrence of BSIs, even among patients treated with lower CS doses. It is unclear whether these observations are confounded by other variables associated with remdesivir use, and intravenous applications of the drug, or may indeed represent a true phenomenon, due to similarities of remdesivir metabolites to adenosine, which is known to attenuate the inflammatory and immune responses [53,54].

The limitations of our research include the retrospective study design and single-center experience. Whether other confounding factors, such as the presence of central venous catheters or an ICU stay, had an impact on BSI could not be determined. Very small proportions of patients received corticosteroids in the dexamethasone 6 mg equivalent dose (22 patients, 1.4%) and 12 mg equivalent dose (8, 0.5%), precluding any meaningful analysis that would be comparable to some previous clinical trials. Also, we could not adequately control for prior chronic corticosteroid exposure, because, as a referral center, our institution received patients who had sometimes already started corticosteroid treatment, due to COVID-19, in various doses before hospitalization in our institution. Our medical records only contained information on the medications used at the time of admission and during the hospital stay. Thus, we did not specifically exclude patients with autoimmune/rheumatic conditions or transplanted organs who might be exposed to corticosteroids for longer time periods prior to admission. Our study encompasses a long time period, characterized by different dominant viral strains and different exposures to vaccination, which we could not adequately control, and which might have effects on the incidences and profiles of BSIs. However, our study is a large real-life cohort representing COVID-19 patients with severe and critical forms of the disease. Data were obtained in a tertiary-level hospital, specialized for the treatment of COVID-19 patients, and might not be generalizable to other levels of care.

## 5. Conclusions

Patients with severe and critical clinical presentations often received high and very high corticosteroid doses in a real-life setting. Their use is progressively associated with a higher risk of bacteremia, independently of sex, comorbidity burden, and mechanical ventilation use. Therefore, clinicians should be more careful when considering higher corticosteroid doses.

## Figures and Tables

**Table 1:** Overview of patients’ comorbidities.

Comorbidity	*n* = 1558
Arterial hypertension	966 (62%)
Diabetes mellitus	454 (29.1%)
Hyperlipoproteinemia	337 (21.6%)
Obesity	600 (38.5%)
Active smoking	96 (6.2%)
Chronic heart failure	135 (8.7%)
Atrial fibrillation	176 (11.3%)
Chronic kidney disease	98 (6.3%)
Chronic liver disease	29 (1.9%)
Chronic obstructive lung disease	85 (5.5%)
Asthma	62 (4%)
Active malignancy	94 (6%)
Autoimmune/rheumatic disease	64 (4.1%)
Dementia	128 (8.2%)
Transplanted organ	19 (1.2%)

**Table 2:** Multivariate logistic regression model investigating the adjusted association of corticosteroid dosing level with the occurrence of bacteremia.

	OR with 95% CI	*p* Value
No corticosteroids Low-dose High-dose Very high-dose	Reference category - OR 1.85, 95% CI (1.21–2.82) OR 1.96, 95% CI (1.12–3.44)	- 0.004 * 0.017 *
Age (years)	OR 1.02, 95% CI (0.99–1.04)	0.123
Male sex	OR 1.73, 95% CI (1.16–2.58)	0.008 *
Charlson comorbidity index	OR 0.88, 95% CI (0.79–0.99)	0.046 *
Mechanical ventilation	OR 18.9, 95% CI (11.9–30.03)	<0.001 *

* statistically significant at level *p* < 0.05.

**Table 3:** Profile of bacterial blood stream infections regarding corticosteroid dosing level.

BSI Profile	No CS (N = 167)	Low-Dose CS (N = 710)	High-Dose CS (N = 539)	Very High-Dose CS (N = 142)	*p* for Trend	*p* for Difference
Positive blood cultures	6 (3.6%)	38 (5.4%)	101 (18.7%)	33 (23.2%)	<0.001 *	<0.001 *
Gram-negative bacteria	5 (3%)	24 (3.4%)	72 (13.4%)	27 (19%)	<0.001 *	<0.001 *
Gram-positive bacteria	4 (2.4%)	16 (2.3%)	53 (9.8%)	18 (12%)	<0.001 *	<0.001 *
Both Gram-positive and -negative bacteria (polymicrobial)	3 (1.8%)	8 (1.1%)	28 (5.2%)	11 (7.7%)	<0.001 *	<0.001 *
*Acinetobacter baumannii*	3 (1.8%)	17 (2.4%)	59 (10.9%)	18 (12.7%)	<0.001 *	<0.001 *
*Staphylococcus aureus*	0 (0%)	3 (0.4%)	24 (4.5%)	4 (2.8%)	<0.001 *	<0.001 *
*Enterococcus faecalis*	1 (0.6%)	3 (0.4%)	17 (3.2%)	3 (2.1%)	0.002 *	<0.001 *
*Enterococcus faecium*	1 (0.6%)	6 (0.8%)	7 (1.3%)	1 (0.7%)	0.585	0.783
*Coagulase negative* *Staphylococcus*	1 (0.6%)	9 (1.3%)	19 (3.5%)	8 (5.6%)	<0.001 *	0.001 *
*Klebsiella pneumoniae*	2 (1.2%)	7 (1%)	10 (1.9%)	4 (2.8%)	0.097	0.314
*Pseudomonas aeruginosa*	2 (1.2%)	2 (0.3%)	3 (0.6%)	2 (1.4%)	0.613	0.271
*Corynebacterium species*	0 (0%)	0 (0%)	3 (0.6%)	3 (2.1%)	<0.001 *	0.002 *
*Escherichia coli*	1 (0.6%)	2 (0.3%)	5 (0.9%)	3 (2.1%)	0.042 *	0.101
*Klebsiella aerogenes*	0 (0%)	0 (0%)	2 (0.4%)	0 (0%)	0.306	0.286
*Proteus mirabilis*	3 (1.8%)	0 (0%)	1 (0.2%)	0 (0%)	0.021 *	<0.001 *
*Stenotrophomonas maltophilia*	0 (0%)	0 (0%)	0 (0%)	1 (0.7%)	0.048 *	0.019 *
*Enterobacter cloacae*	1 (0.6%)	0 (0%)	2 (0.4%)	0 (0%)	0.849	0.265
*Staphylococcus haemolyticus*	0 (0%)	0 (0%)	2 (0.4%)	0 (0%)	0.306	0.286
*Serratia marcescens*	0 (0%)	0 (0%)	1 (0.2%)	1 (0.7%)	0.056	0.176
*Providencia stuartii*	0 (0%)	0 (0%)	2 (0.4%)	0 (0%)	0.306	0.286
*Beta hemolytic Streptococcus*	0 (0%)	0 (0%)	0 (0%)	1 (0.7%)	0.048 *	0.019 *
*Streptococcus pneumoniae*	0 (0%)	1 (0.1%)	0 (0%)	0 (0%)	0.598	0.754
*Bacteroides species*	0 (0%)	0 (0%)	1 (0.2%)	0 (0%)	0.469	0.595
*Haemophilus parainfluenzae*	0 (0%)	0 (0%)	1 (0.2%)	0 (0%)	0.469	0.595
*Providencia rettgeri*	0 (0%)	0 (0%)	1 (0.2%)	0 (0%)	0.469	0.595

* statistically significant at level *p* < 0.05.

## Data Availability

Data can be obtained from the corresponding author per reasonable e-mail request.
